# Examining Health-Related Effects of Refurbishment to Parks in a Lower Socioeconomic Area: The ShadePlus Natural Experiment

**DOI:** 10.3390/ijerph17176102

**Published:** 2020-08-21

**Authors:** Suzanne J. Dobbinson, Jody Simmons, James A. Chamberlain, Robert J. MacInnis, Jo Salmon, Petra K. Staiger, Melanie Wakefield, Jenny Veitch

**Affiliations:** 1 Cancer Council Victoria, Melbourne, VIC 3004, Australia; jodymichellesimmons@gmail.com (J.S.); Jamie.Chamberlain@cancervic.org.au (J.A.C.); Robert.MacInnis@cancervic.org.au (R.J.M.); melanie.wakefield@cancervic.org.au (M.W.); 2 Centre for Epidemiology and Biostatistics, The University of Melbourne, Parkville, VIC 3010, Australia; 3 Institute for Physical Activity and Nutrition (IPAN), School of Exercise and Nutrition Sciences, Deakin University, Geelong, VIC 3220, Australia; jo.salmon@deakin.edu.au (J.S.); jenny.veitch@deakin.edu.au (J.V.); 4 School of Psychology, Deakin University, Geelong, VIC 3220, Australia; petra.staiger@deakin.edu.au

**Keywords:** public health, intervention, parks/trails, neighborhood/community, behavior change, active living, low income

## Abstract

Degraded parks in disadvantaged areas are underutilized for recreation, which may impact long-term health. Using a natural experiment, we examined the effects of local government refurbishments to parks (n = 3 intervention; n = 3 comparison) in low socioeconomic areas (LSEA) of Melbourne on park use, health behavior, social engagement and psychological well-being. Amenities promoting physical activity and sun protection included walking paths, playground equipment and built shade. Outcomes were measured via systematic observations, and self-report surveys of park visitors over three years. The refurbishments significantly increased park use, while shade use increased only in parks with shade sails. A trend for increased social engagement was also detected. Findings infer improvement of quality, number and type of amenities in degraded parks can substantially increase park use in LSEA. Findings support provision of shade over well-designed playgrounds in future park refurbishments to enhance engagement and sun protection behavior. Further research should identify park amenities to increase physical activity.

## 1. Introduction

Physical inactivity exerts significant impacts on disease outcomes for communities [1,2]. Parks in urban areas play a vital role in promoting health [3], providing opportunities for free outdoor recreation, physical activity, contact with greenery, and for increased social interactions among community members across age groups [4,5,6,7,8,9]. Nevertheless, the attractiveness and amenity of parks are generally not equitable, with parks located in socioeconomically disadvantaged areas typically having fewer and/or poorer-quality amenities than advantaged areas [10,11,12,13]. This lack of amenity likely contributes to lower engagement in outdoor recreation and physical activity and to having fewer opportunities for park-related social interaction among residents in these disadvantaged communities. Such disparities in amenities might partly explain the amelioration of higher mortality rates from circulatory diseases in low socioeconomic areas with greater access to green space [14]. Moreover, socioecological models highlight the role of multiple levels of influence of the social and physical environment, including the natural and built environments, on health behavior [15,16,17]. Therefore, refurbishment of parks in disadvantaged areas may provide health-related benefits in addition to redressing disparities in public infrastructure provided in these areas.

The studies that examined the effects of modifying the built environment in parks and/or green space on park usage and park-based physical activity have shown variable impact [14,15,16]. Generally, these studies relied on quasi-experimental designs rather than large-scale randomized or matched comparison trials and are subject to bias [14,15,16]. Many of these studies included both changes to the social environment and to the physical environment in the parks. Thus, further limiting evidence of the effects for modifying the physical environment alone on park use and physical activity. Findings from multiple studies in different contexts may help causal inference. Research is needed to prospectively identify the broader health-related effects of park modifications to examine outcomes beyond their potential to attract greater park use and increase physical activity. Furthermore, in countries where skin cancer incidence and mortality rates are high—such as Australia, which has one of the highest incidence rates of skin cancer globally [18,19,20]—it is particularly important to explore the effects of park modifications on sun protection behavior. Moreover, while the restorative properties of contact with natural environments (greenness) are well-established [21], understudied additional benefits of park refurbishment on psychological well-being, opportunities for social interactions and community engagement may also be realized.

The current study (ShadePlus) aimed to assess the impact of refurbishments, including provision of built shade, walking paths and high-quality playgrounds, to parks located in a lower socioeconomic area of Melbourne, Australia. We hypothesized that refurbishing existing parks to include the above key amenities would increase park use, physical activity and the use of shade in the parks. Further, we anticipated that the increase in park use would promote opportunities for social and community engagement, and through contact with open space and greenery, may have benefits for psychological well-being.

## 2. Methods

We used a natural exposure experiment to examine the broad health-related effects of the ShadePlus park refurbishments at three intervention parks relative to three comparison parks (not receiving refurbishment) during spring and summer over three consecutive years, i.e., at pre-test (T1: 2013–2014), at post-test in the first year after the refurbishment (T2: 2014–2015), and at follow-up (T3: 2015–2016). The methods are briefly described below. The study protocols are published and include further details on the parks selected, park amenities, and measures provided [22]. This study was approved by Cancer Council Victoria’s Human Research Ethics Committee (HREC 1311).

### 2.1. Study Sites

Brimbank City Council, a local government area with a program of planned park refurbishments and located in one of the lowest socioeconomic areas of Melbourne [23], partnered the project. The area is in the outer western suburbs of the city, which are prone to drought, with heavy clay soils and less than 10% tree canopy cover [24]. These suburbs have a culturally diverse population of approximately 185,000 residents in 2009 [23]. In consultation with the council’s urban design manager, parks pre-scheduled to receive refurbishments and comparison parks (matched for extent and condition of amenities, park size, residential housing type and densities in surrounding suburbs and demographics) were selected for the study. Approximate matching was achieved on the number and type of amenities; however, two of the comparison parks were 2.5 to 4.2 times smaller than any of the intervention parks.

**Figure 1:**
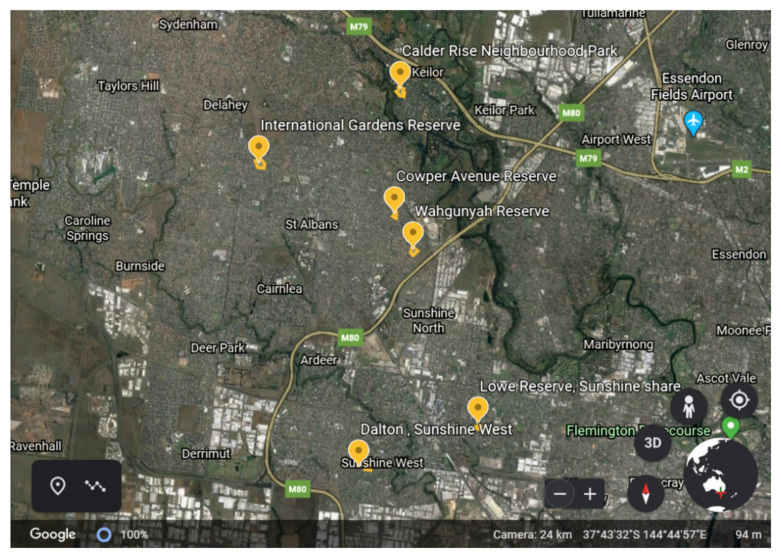
Locations of six study parks in north west Melbourne, Victoria, Australia—source Google Earth: Google, 2020.

The locations of the study parks are presented in Figure 1. The catchment suburbs are between 11 and 23 km north west of Melbourne CBD [25]. Intervention parks were Dalton Reserve, Wahgunyah Reserve and Calder Rise Reserve (recently renamed as Calder Rise Neighbourhood Park). Comparison parks were Lowe Reserve, Cowper Crescent Reserve and International Gardens. The characteristics of each park were previously published [22], including park size (5650–28,645 m^2^), park catchment’ population, density, median age of residents and household income. The median age of residents (34–37 y.) and weekly household income levels in the suburb surrounding each park were relatively comparable (AUD 865–986), with one outlier suburb where the park was adjacent to a natural ravine and creek bed. This suburb had, on average, slightly older residents (median 43 y.) with higher weekly household incomes (median AUD 1353) compared with the suburbs of the other study parks. Nonetheless, this park catchment is subject to the broader socioeconomic disadvantage of the district, with Brimbank City Council Area being ranked the fourth lowest local government area in the state of Victoria [25].

### 2.2. Intervention

**Figure 2:**
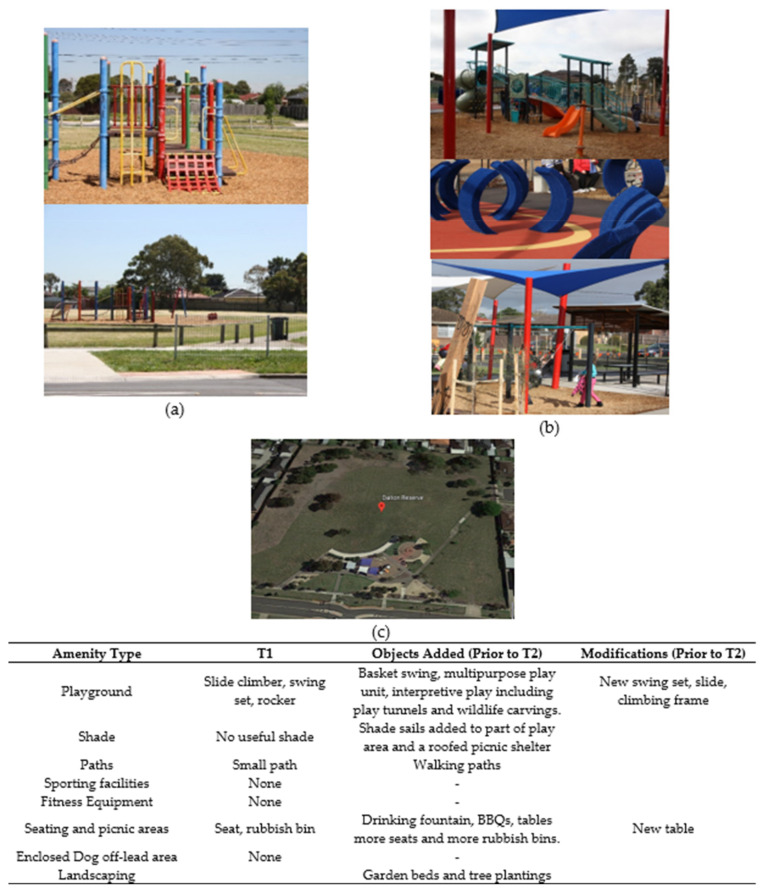
Dalton Reserve’ amenities. (**a**) Playground area and walking path before refurbishment (T1). (**b**) Playground areas and seated areas after refurbishment (T2–T3). (**c**) Satellite image of park after refurbishment (downloaded from Google Earth: Google, 2020).

**Figure 3:**
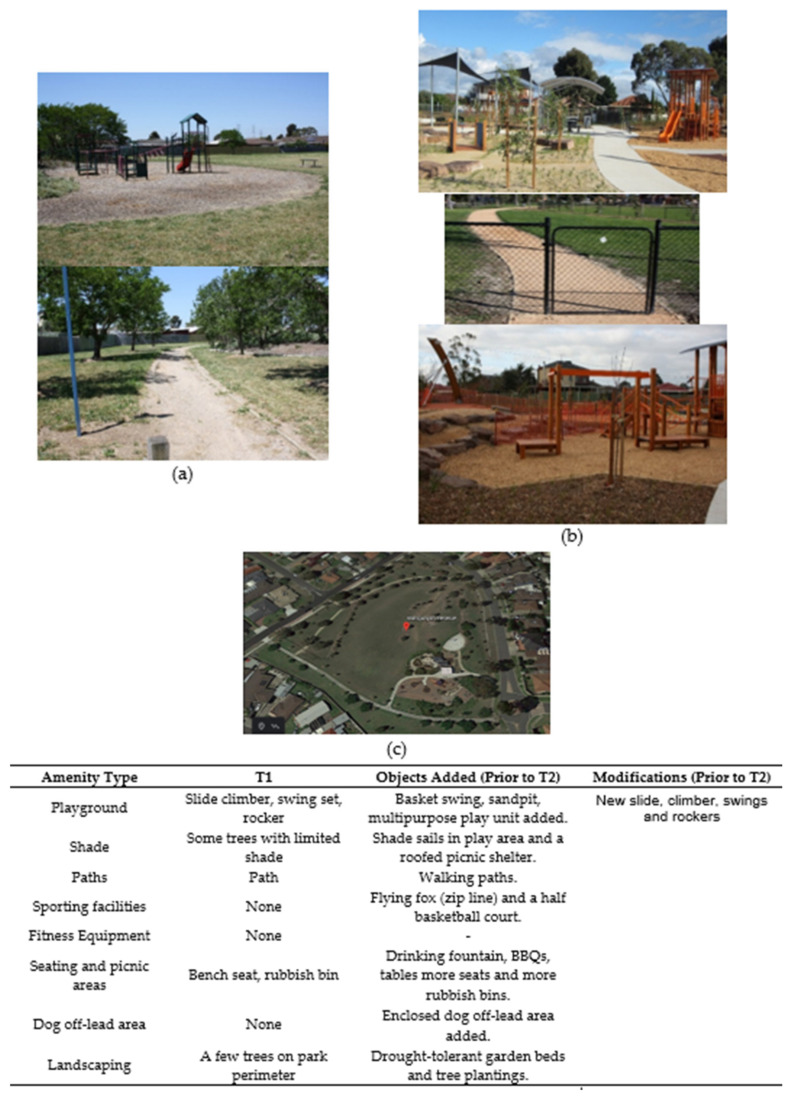
Wahgunyah Reserve’ amenities. (**a**) Playground area and walking path before refurbishment (T1). (**b**) Playground areas, seated areas, walking path and enclosed dog off-lead area after refurbishment (T2–T3). (**c**) Satellite image of park after refurbishment (downloaded from Google Earth: Google, 2020).

**Figure 4:**
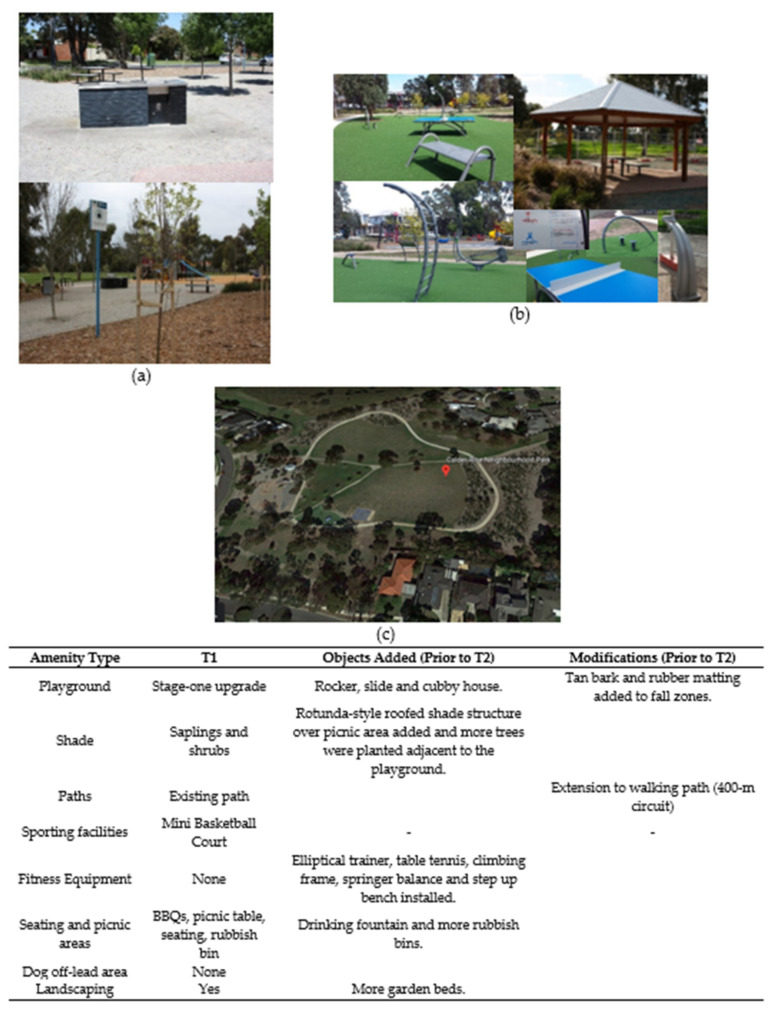
Calder Rise Reserve’ amenities. (**a**) Picnic area and playground before refurbishment (T1). (**b**) New fitness area and picnic rotunda after refurbishment (T2–T3). (**c**) Satellite image of park after refurbishment (downloaded from Google Earth: Google, 2020).

**Figure 5:**
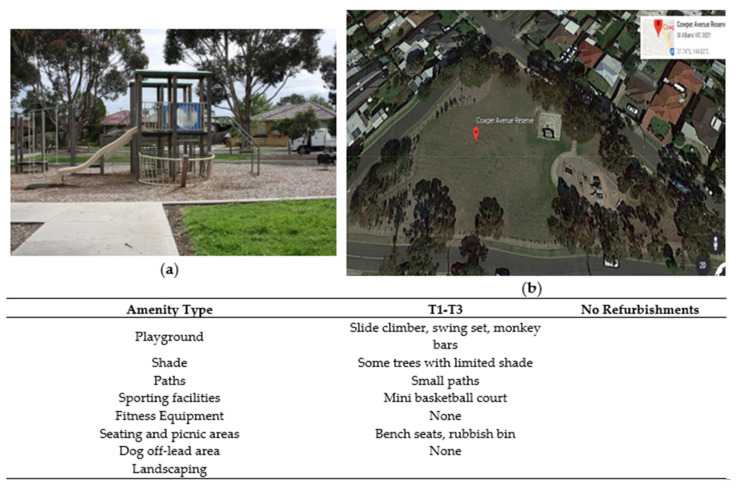
Cowper Reserve amenities. (**a**) Part of playground at T1. (**b**) Satellite image of park (Google Earth image: Google, 2020).

**Figure 6:**
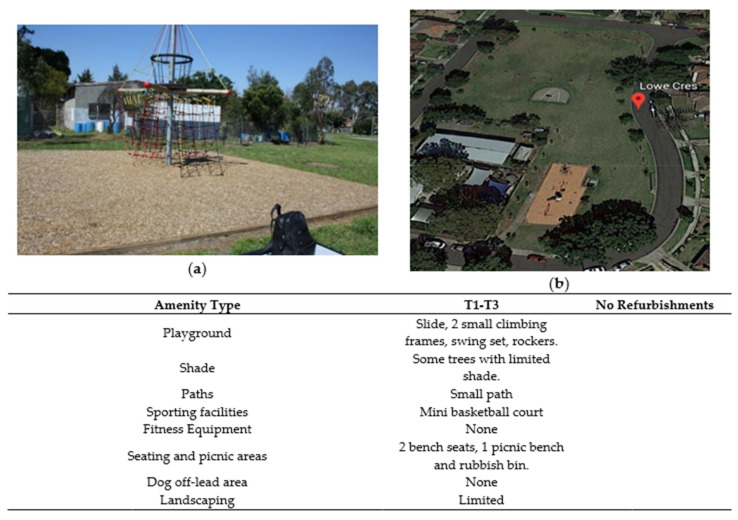
Lowe Crescent Reserve’ amenities. (**a**) Part of playground area T1. (**b**) Satellite image of park (downloaded from Google Earth: Google, 2020).

**Figure 7:**
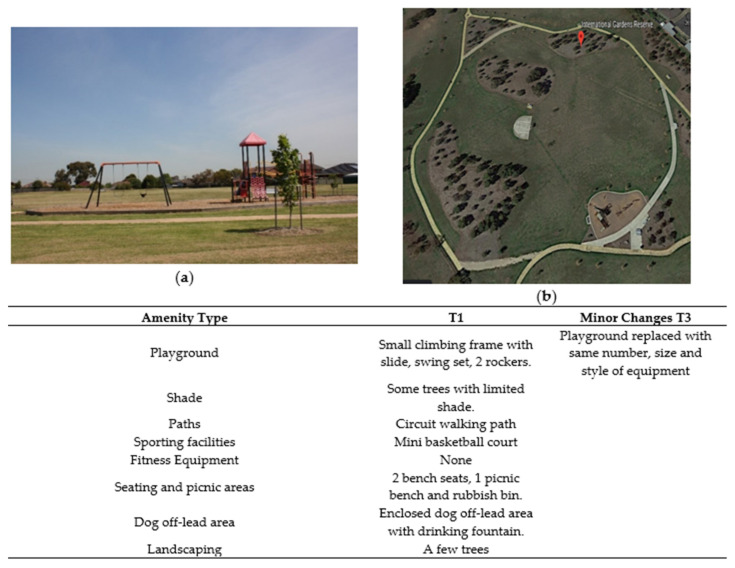
International Gardens’ amenities. (**a**) Playground area T1. (**b**) Satellite image of park showing recent tree plantings (downloaded from Google Earth: Google, 2020).

Following discussion with the urban designer, planned refurbishments were refined where feasible to include features that evidence suggests might promote park-based physical activity (age-appropriate playground equipment and quality walking paths) and sun protection (built shade including a shade-sail for the children’s playground) [26,27]. Two of the intervention parks (Dalton Reserve and Wahgunyah Reserve) received all desired intervention elements including new playground equipment with a shade sail and new walking paths. The other intervention park (Calder Rise Reserve) did not receive the desired shade sail over the playground and gained only minor playground amenities (i.e., resurfacing with tan bark and rubber fall zones, an additional slide, a cubby or club house and playground rocker). This park had previously received new playground equipment, so this refurbishment provided built shade for seating and picnic areas (a wooden rotunda), a circuit walking track, fitness equipment, and tree saplings next to the playground. Photographs of each intervention park before and after the refurbishments are provided in Figure 2, Figure 3 and Figure 4, while comparison parks amenities remained largely unchanged across the study (Figure 5, Figure 6 and Figure 7). Open days were held at Dalton Reserve and Wahgunyah Reserve during mid-winter (July 2014) on completion of the park refurbishments. An open day at Calder Rise Reserve had been provided with the partial refurbishments completed prior to the study. These events included an official opening, face painting, and a barbeque lunch. No other marketing or recreation programs were held at the parks.

### 2.3. Outcomes

The primary outcomes assessed included: (1) the number of people observed in the park; (2) the number of people observed engaging in active recreation (defined as moderate-to-vigorous physical activity); and (3) the number of people observed using shade. The secondary outcomes were self-reported during intercept interviews in the park and included: emotional state of park visitors (measured by items from the Positive and Negative Affect Schedule (PANAS) emotional state scale [28,29]); social connectedness (number of times met or talked with new (unacquainted) people and known people at the park, and participation in social events at the park); perceived community engagement scores of park visitors; and perceived acceptability of the park refurbishments (as measured by the mean ratings of the aesthetics of park amenities).

### 2.4. Procedure

The observations and intercept surveys were conducted by pairs of research staff at the parks during T1, T2, and T3. Observations of the playground and ‘rest of park’ at each park were scheduled for eight randomly selected days across each test period (one weekend day and one weekday per month across 4 months). Each observation day comprised a total of 11 × 30-min scans of the playground and rest of park between 7:00 and 18:30. All observation measures were completed as planned except for the last 30-min observation scan on one date during T3 at one of the parks. 

At the end of each observation shift, and for several weeks in early March, research staff approached park visitors and invited them to complete a brief intercept survey that included questions about their activities at the park, emotional state, opportunities for socializing at the park, perceived aesthetics of amenities and perceptions of their connectedness to the local community. Eligible participants spoke English and were 14 years or older [22].

The ambient temperatures and precipitation during the study are described in Appendix A, Table A1. Temperature conditions were variable (7.9 to 39.8 °C), but with consistent daily fluctuations and a similar range of temperatures for each test period. There was little rainfall during the study observations.

### 2.5. Statistical Analysis

Descriptive statistics were used to assess the equivalence of park visitor counts, visitor demographic characteristics, timing of park use, observed cloud cover [30] and wind strength between intervention and comparison parks during T1.

The primary analyses were largely conducted according to the published analysis plan [22] using cluster-level analyses treating parks as clusters, which is considered the most robust approach to assess impact for studies with a small number of clusters [31]. Intervention impact was examined both for intention to treat (all six parks) and an analysis of effects by intervention received (excluding Calder Rise Reserve and abandoning the pair-matched design). 

Mean counts of park visitors, people using shade and engaged in moderate-to-vigorous physical activity were plotted for each study group (i.e., intervention or comparison) and study period (i.e., T1, T2 or T3). Analyses accounted for clustering within parks using a single cluster count for each outcome and park during the study period. This involved aggregating observed counts for each of the playgrounds and the rest of the park, 11 × 30-min observation scans per date, and eight observation dates at each park for each study period.

The primary analyses were based on t-tests comparing the group difference in mean change in outcomes from T1 to T2 for the intervention parks and comparison parks. The sustainability of effects was assessed using t-tests comparing group mean change in outcomes from T1 to T3. Effect sizes, measured by Cohen’s d, and 95% CI were calculated. The ratio of change from T1 to T2 between intervention and comparison parks was calculated using the geometric average visitor counts at each study period for each group.

Secondary outcomes (self-report measures) were analyzed using t-tests to compare the intervention and comparison parks using the change in mean scores for each park from T1 to T2 and T1 to T3. 

The analyses were performed using Stata 14.2 (StataCorp, College Station, TX, USA) and Microsoft Excel. Missing data were excluded from all analyses. 

The data are publicly available from the openICPSR repository [32].

## 3. Results

A count of 1670 people used the six study parks during T1, equivalent to an average of three people observed per park during each 30-min scan (between 07:00 and 18:30). The greatest number of park visitors were observed during the afternoons (16:00 to 18:30) for both study groups. Park visitors comprised mostly young adults (20–49 years) and children (<14 years). At pre-test, there were few variations in patterns of usage by study group (refer Table 1). A slightly higher proportion of females and children at intervention parks relative to comparison parks (44.7% cf. 37.1%; and 35.9% cf. 30.3%, respectively) and a slightly higher proportion of visitors were observed at intervention parks during times of strong wind relative to comparison parks. Additionally, there was a greater proportion of park visitors engaged in moderate- or vigorous-intensity physical activity at the intervention parks compared with the comparison parks during T1 (86.6% cf. 74.5%). At each time point, there was a high percentage of observation scans with no people in the park (T1: 26%, T2: 17%, T3: 20%).

### 3.1. Intervention Impact

**Figure 8:**
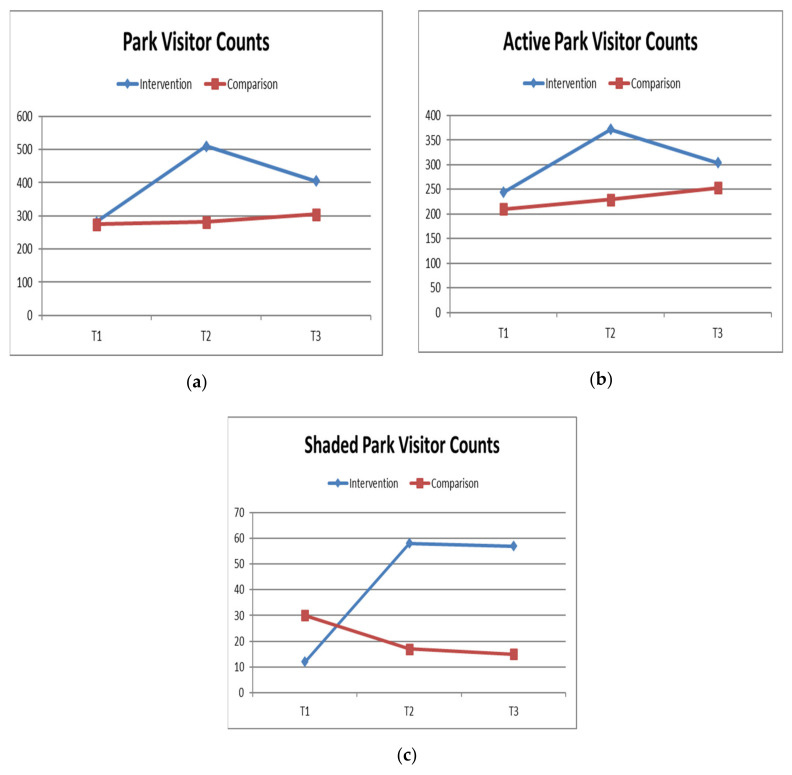
Outcomes over time by study group including all six study parks. (**a**) Change in mean counts of park visitors at intervention and comparison parks across the study; (**b**) Change in mean counts of park visitors who were moderately or vigorously active at intervention and comparison parks across the study; (**c**) Change in mean counts of park visitors who were using shade at intervention and comparison parks across the study.

Figure 8a–c describe the mean counts of park visitors, physically active visitors, and those using shade at intervention parks and comparison parks at each time period. A large increase from T1 to T2 was apparent in the mean number of park visitors (T1: x = 282, SD 228; to T2: x = 510, SD 143) and those physically active (T1: x = 244, SD 195; to T2: 372, SD 200) at intervention parks, compared with a small increase in park visitors (T1: x = 275, SD 86; to T2: x = 282, SD 63) and those physically active (T1: x = 210, SD 63; to T2: x = 230, SD 75) at comparison parks. This higher level of park use and physical activity at intervention parks from T1 to T2 was somewhat reduced by follow-up (T3). The mean number of shaded visitors was low at T1 in both groups (Ix: x = 12, SD 20; C: x = 30, SD 32). Shade use increased in intervention parks at T2 (x = 58, SD 41), and was steady at T3 (x = 57, SD 64), but decreased slightly in the comparison parks (T2: x = 17, SD 17; to T3: x = 15, SD 8). 

The results of the intention-to-treat primary analysis (Table 2) demonstrate the mean increase in park visitors from T1 to T2 was greater at intervention parks relative to comparison parks (Ix: x = 273, SD 72.1; C: x = 7.3, SD 31.5, *p* = 0.02; Cohen’s *d* = 7.0, 95% CI 2.0 to 12.0). This change represents over a two-fold increase from T1 to T2 in visitor counts at intervention parks (ratio of change 2.24) relative to comparison parks (ratio of change 1.05). However, the change in park use was not sustained into T3 (T1 to T3: p = 0.31, Cohen’s d = 4.3, 95% CI −6.1 to 14.8). There was no appreciable difference in mean change from T1 to T2 in the number of visitors engaging in moderate-to-vigorous physical activity, and the number of visitors using shade by study group (*p* = 0.15 and *p* = 0.15, respectively).

Results of intervention received, which excluded data from Calder Rise Reserve, showed a difference in mean change in visitor counts at intervention parks relative to comparison parks between T1 and T2 (*p* = 0.01, Cohen’s d = 8.4, 95% CI 3.9 to 12.9) and between T1 and T3 (*p* = 0.002, Cohen’s *d* = 8.1, 95% CI 5.6 to 10.6). This analysis also showed a difference in the mean change in shaded visitor counts at intervention parks relative to comparison parks from T1 to T2 (*p* = 0.04, Cohen’s *d* = 5.8, 95% CI 0.4 to 11.1).

Intercept surveys of park visitors were collected at each time-period (T1: n = 88; T2: n = 103; T3: n = 66). There was no evidence of intervention impact for PANAS emotional state, community social cohesion or perceived attractiveness of the parks (Table 3). There was some evidence (marginal significance) for an increase in social interactions with unacquainted people at the intervention parks from pre-test to follow-up, with the difference in mean group change from T1 to T3 greater at intervention parks than comparison parks (Ix: x = 0.39, SD 0.9; C: x = −1.2, SD 0.6, *p* = 0.05).

### 3.2. Descriptive Outcomes for Individual Parks

**Figure A1:**
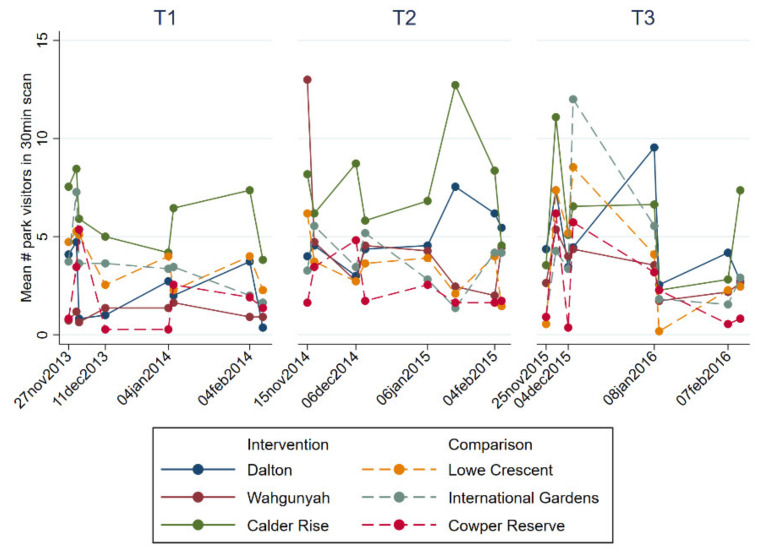
This figure describes the mean visitor counts per 30-min observation scan at each park aggregated by observation date over time.

Table 4 describes the observed numbers of park visitors, those engaged in moderate or vigorous physical activity and those visitors using shade at individual parks during each study period. During T1 there was considerable variation between parks in the numbers of park visitors, active visitors and the number of visitors shaded. However, the number of park visitors and the number of visitors engaged in moderate or vigorous-intensity physical activity were consistently highest at Calder Rise Reserve (Intervention Pair C) at each time point. In contrast, the lowest number of park visitors and lowest number of visitors engaged in moderate or vigorous physical activity were observed at Wahgunyah Reserve (Intervention Pair B) at T1. Park visitor counts increased substantially from T1 to T2 in all three intervention parks, with small increases from T1 to T2 also observed for all three comparison parks. Park visitor counts decreased from T2 to T3 at all three intervention parks, but at Dalton Reserve to a lesser extent (Intervention Pair A). Concurrently, park visitor counts at comparison parks appeared to increase slightly from T2 to T3. Additionally, analysis of the change in mean visitor numbers during 30-min observation scans at each park over time (refer to Appendix B, Figure A1) demonstrated the extent of variability in park use on different dates at each of the parks within each study period. A higher variation in mean daily park use per 30-min observation scan was observed at T2 and T3 among the intervention parks relative to the comparison parks. This included single dates with unusually high mean park use per 30-min observation scan for the time period at a given intervention park.

Only a small number of park visitors were observed using shade at the intervention parks during T1. Shaded visitor counts increased substantially from T1 to T2 in two of the intervention parks (Dalton Reserve and Wahgunyah Reserve) but decreased in the other intervention park (Calder Rise Reserve). The number of park visitors using shade increased again from T2 to T3 at Dalton Reserve, while decreasing at the other two intervention parks during this period. Shade use at the comparison parks was more variable across the three study periods.

## 4. Discussion

The findings of the ShadePlus study contribute to evidence supporting the role of physical changes to the park environment in attracting more visitors to parks in socioeconomically disadvantaged areas [33,34,35]. The study found more visitors used the refurbished parks than the comparison parks, particularly in the first spring-summer after refurbishment. There was a 124% increase in mean park use at the intervention parks from T1 to T2 relative to a 5% increase at comparison parks. Changes in shade use and physical activity were not apparent on an intention-to-treat basis; however, analyses that excluded the intervention park that did not install shade sails over the playground showed an increase in shade use at the other intervention parks from T1 to T2 and increased park use sustained into a second year (T3) compared with the comparison parks. It is likely that this positive effect on shade use was in-part related to the installation of shade sails over the well-designed playgrounds, in addition to providing the roofed shade at picnic areas. Provision of shade in areas with the most utilized amenities may also be a factor. There was an increase in the mean number of park visitors engaging in moderate-to-vigorous physical activity at intervention parks from T1 to T2, but the change was not statistically different relative to comparison parks. Interestingly, a trend for increased social engagement with unacquainted people at the intervention parks occurred much later than the change in park usage (i.e., from T1 to T3), suggesting a delay in opportunities for social engagement after the initial increase in visitor numbers at these parks. However, no overall improvements in park visitors’ self-reported emotional state occurred at the intervention parks compared with comparison parks.

The findings for individual parks further suggest considerable variability in impact of refurbishments on park use and physical activity outcomes between the different intervention parks. The refurbishment of the intervention park with the lowest park use (Wahgunyah Reserve), and little more than basic playground facilities at T1, attracted the greatest number of visitors at T2 (338% increase), which marginally decreased at T3 although remaining 203% above baseline levels. In contrast, the stage-two park refurbishment had a very modest increase from T1 to T2 (26%) and a decrease at T3 with visitor numbers reducing to 7% below baseline, suggesting novelty may have been a factor with the initial increase. A similar pattern of change was observed for counts of visitors engaged in moderate-to-vigorous activity at these parks. The initial condition and type of amenities at the parks, as well as the quality and type of amenities in the refurbishments may partially explain the variation in initial and longer-term use of these parks. Wahgunyah Reserve refurbishments included a zip line and a half basketball court to promote activity among adolescents and young adults, as well as providing a large picnic and BBQ area. The fitness equipment and other amenities in the stage two refurbishment at Calder Rise Reserve appear insufficient to have attracted more visitors to this park which already had relatively high visitor numbers. However, the study design limits the extent to which these specific refurbishment components attracted the sustained increase in visitors to these parks.

Although commonly studies evaluating effects of park refurbishments on physical activity and park use have been conducted alongside recreation programs (e.g., park-based physical activity programs) [33,35] or community engagement [36], few have studied the effects of park refurbishment alone [33,34,35]. Two of these studies involving park refurbishment alone were also set in lower socioeconomic areas of Melbourne. The studies examined the impact of installing a play-scape [34] and a playground, walking path and dog off-leash area [37] and found an increase in visitor numbers and visitors engaging in park-based physical activity following park refurbishment [34,37]. Another study set in parks in lower-socioeconomic communities in the U.S. also found that renovations to sports playfields alone increased visitor numbers and physical activity of visitors [38]. The studies are not directly comparable with ShadePlus findings given the different observation times, park attributes, condition and type of amenities before and after refurbishment. All four studies found an increase in park usage in the first year following refurbishment, albeit ranging from a two-fold increase for ShadePlus compared with a five- to eight-fold increase in the other studies. However, structural changes alone did not always result in substantial increases in physical activity levels of park visitors, as observed in ShadePlus and a study [8] of fitness zones added to 12 parks in the U.S. Similarly, increased park use, but no change in physical activity, was observed following the building of new pocket parks in areas with a high rate of poverty in the U.S. [39]. However, this was attributed to the small size of the parks (<1 acre), which limited scope to include walking paths but provided an important venue for social interactions and children’s play [39]. The parks in our study were all larger than one acre in size with enough space for walking paths, basketball courts and other facilities to promote physical activity. In fact, a high proportion of visitors observed using the study parks (86.6%) were moderately or vigorously active at T1. However, baseline park utilization was overall relatively low prior to the refurbishments with scope to attract more residents to use the parks for physical activity. The average total visitor counts per park were 278 visitors for Shadeplus at T1, while pretest visitor counts for the other refurbishment alone studies were an average of 136 visitors for three pocket parks [39], 608 visitors in 12 parks in the fitness zone study [8], and 2378 visitors for two large parks in the Melbourne playscape study [34]. Although these studies [8,34,39] varied by park size, amenities, number of target areas and time-of-day observed, they had a similar number of days of observation (7–8 days) per study period and may provide at least some indication of relative utilization.

These findings suggest that further research to identify how to sustainably increase park-based physical activity for different communities is worth pursuing. Moreover, studies have found park refurbishments a cost-effective way to increase physical activity [40]. Innovative amenities may be required. For example, the addition of musical stairs in a college was shown to stimulate students to use the stairs by making it fun [41]. Thus, identifying novel park amenities that people find attractive to try and enjoyable when experienced may be valuable in promoting physical activity. Considering two of the Shadeplus parks added innovative amenities (fitness zones and a flying fox), there may be benefit in examining the change in physical activity levels for specific parks in future analyses. Nonetheless, parks that are well maintained, safe and provide shade are rated highly by park visitors as important for promoting physical activity [42].

Our findings for increased shade use at the refurbished parks with shade sails are consistent with previous research on adding built shade to passive recreation areas in 23 secondary schools in Melbourne, Australia [26], and to 36 parks in Denver, U.S. and Melbourne, Australia [27]. These studies included a number of schools and parks located in lower socioeconomic areas. This is also in line with a park development study where a well-used shade tree in a garden space was renovated into a shady seated area as part of an urban greenspace development in Denver, U.S. [43]. The shade tree was anecdotally highly valued and used for shade and socializing by the local community prior to the development, whilst post construction it continued to attract a large proportion of park visitors. Our study more specifically evaluates the role of creating new shaded areas to the park environment on shade use. Given a large shade tree takes many years to grow, establishing that built shade attracts people to use rather than avoid shade is important. Built shade may be needed in parks as an interim strategy, or the only feasible strategy in arid areas, for reducing park visitors’ exposure to ultraviolet radiation.

A key strength of this study is that it prospectively evaluated the effects of modifying the park environment on sun protection, emotional state, and social connectedness alongside measures of park use and physical activity levels. The study’s strengths include the use of objective primary measures and the frequency and extended period over which they were measured, three time periods across 28 months, enabling the assessment of long-term outcomes. All observations were conducted at the same times and dates to minimize different contexts and weather conditions as potential confounders of effects. Additionally, the intervention parks were selected from parks pre-scheduled to receive refurbishment and the comparison parks were matched as close as possible to minimize selection bias.

Study limitations included a relatively small number of parks with which to assess the impact of the refurbishments, resulting in sensitivity to detect only large differences in outcomes. Moreover, there was increased spread in daily numbers of people observed using individual study parks from T1 to T2 and from T2 to T3, and increased park use in the comparison parks from T2 to T3, which also limited sensitivity to detect an intervention effect. There was a high proportion of observation data where shade use was unobservable on overcast days. We expect that there would be more shade use on sunny days, so it is unlikely that these missing shade observations on overcast days would have revealed substantially more shade use at intervention parks. There was considerable variability in daily and hourly temperatures during the park observations, which likely influenced use of the parks, playground equipment and shaded areas. This potential bias of intervention effects was minimized by conducting observations for all parks on identical dates and the same seasons for each test period. Temporal weather changes might still be a source of confounding, given slightly higher temperatures occurred during T3. Temporal variation in the population characteristics of the residents residing near the parks may also be a significant source of confounding for these types of intervention. Census information on the park catchment populations were available at a four-yearly interval, so this potential variability could not be adequately assessed. There were no significant housing developments near the parks during the study. Five of the study parks were located in suburbs with comparable weekly household incomes of residents and similar socioeconomic scores [44]. Our sensitivity analyses excluded one park located in a suburb with relatively higher household expenditure and a higher area-based socioeconomic score. This park also received an initial playground refurbishment prior to the study. Therefore, the five-park analysis provides a more robust analysis of intervention effects for a refurbishment of degraded parks with limited quality and number of amenities. Nonetheless, park refurbishments typically involve an array of components. Thus, studies that build knowledge of effects for a mix of components and levels of refurbishment can be informative. For policy reasons it may be useful in future analyses to also examine intervention effects stratified by specific observation conditions (e.g., sunny/cloudy, week day/weekend, time of day); and by the characteristics of the park visitors (e.g., age-group and sex). For example, a high proportion of children and young adults were using the parks at T1 and it would be interesting to identify whether the refurbishments attracted different age groups to use the parks to supplement other recent studies [45,46]. Moreover, if people perceived the refurbished parks to be more attractive for picnics and social gatherings than prior to refurbishment, we might expect to see high numbers of visitors at a given park on specific dates. Although this pattern of use was observed at intervention parks, future studies might consider ways to measure these activities during the observations to better explain patterns of use at individual parks. Further it is important to ensure that local residents’ preferences and opinions are sought on desirable amenities during the planning phase for such refurbishments, particularly in low income areas, but also alongside these intervention studies.

Our analyses of secondary outcomes were also limited by the low number of park intercept surveys completed. These results are, therefore, indicative and cannot assess small effects.

Despite our limited power in precluding an examination of the effects of refurbishments on visitors’ psychological well-being (emotional state), there is considerable evidence of the benefits of contact with nature and green space on psychological well-being [6,7,47,48]. Completion of a larger number of intercept interviews in future park refurbishment studies would more rigorously test potential benefits on well-being and other outcomes.

Although myriad ecological studies suggest the benefits of parks and green space on physical activity and health [3,4,9,33], studies such as this natural experiment can contribute to building causal inference and help to advocate for future investment in park refurbishment. This study highlights the benefits to be gained by public health researchers collaborating with municipalities and urban designers to identify opportunities to test and improve health-related outcomes of infrastructure changes. These projects provide a platform for dialogue about the evidence for specific amenities likely to be beneficial for physical activity and other health-related outcomes, and in return offer valuable data on outcomes of development projects for local communities from which cost effectiveness may be assessed and the design of future projects refined.

## 5. Conclusions

The positive impact of the ShadePlus park refurbishments on park use, and evidence of effects on shade use and possibly social connectedness in some of the refurbished parks, suggest that improvement of the quality, number and type of amenities at degraded parks in low socioeconomic areas may have substantial benefits for improving participation in recreation at parks, contact with green space and social interaction in these communities. Provision of shade in refurbishments at parks, particularly in jurisdictions with high skin cancer rates, may assist in reducing potential harm from increased exposure to ultraviolet radiation during outdoor recreation. However, as ShadePlus park refurbishments showed no significant effects on physical activity, further research is needed to reveal the specific park amenities that may have the largest impact on park-based physical activity.

## Figures and Tables

**Table 1:** Observed number of park visitors according to demographic characteristics, observation times and days, and weather at T1. (total person counts ^a^).

Variable	Intervention	Comparison	Total
	*n* (%)	*n* (%)	*n* (%)
Total number of visitors ^b^	846 (100)	824 (100)	1670 (100)
Males	464 (54.9)	510 (61.9)	974 (58.3)
Females	378 (44.7)	306 (37.1)	684 (41.0)
Undetermined	4 (0.5)	8 (1.0)	12 (0.7)
Age ^c^			
Child < 14 years	303 (35.9)	248 (30.3)	551 (33.2)
Adolescent 14–19 years	85 (10.1)	151 (18.5)	236 (14.2)
Adult 20–49 years	305 (36.1)	284 (34.7)	589 (35.4)
Adult 50 + years	151 (17.9)	135 (16.5)	286 (17.2)
Activity levels			
Lying/sitting	63 (7.5)	107 (13.1)	170 (10.2)
Standing	44 (5.2)	83 (10.1)	127 (7.7)
Walking/moderate	587 (69.9)	478 (58.3)	1065 (64.2)
Vigorous	146 (17.4)	152 (18.5)	298 (18.0)
Active	733 (86.6)	630 (74.5)	1363 (81.6)
Time of day			
7am–8:30am	158 (18.7)	116 (14.1)	274 (16.4)
11:30am–1:00pm	194 (22.9)	114 (13.8)	308 (18.4)
4:00pm–6:30pm	494 (58.4)	594 (72.1)	1088 (65.2)
Day of week			
Weekdays	460 (54.4)	351 (42.6)	811 (48.6)
Weekends	386 (45.6)	473 (57.4)	859 (51.4)
Cloud cover			
Clear skies/thin cloud	343 (40.5)	348 (42.2)	691 (41.4)
Cloudy	503 (59.5)	476 (57.8)	979 (58.6)
Wind			
None–slight	275 (32.5)	286 (34.7)	561 (33.6)
Moderate	382 (45.1)	422 (51.2)	804 (48.1)
Strong–very strong	189 (22.3)	116 (14.1)	305 (18.3)

^a^ Total person counts represent counts of individuals observed during eleven 30-min observation scans per date on eight dates during the study period overall and by sex, age and observation features. These data were aggregated by park and study group. ^b^ The mean number of visitors during a 30-min scan were Intervention: 3.20 (SD = 4.06); Comparison 3.12 (SD = 4.21); Total 3.16 (SD = 4.13). ^c^ Age excluded n = 8 person counts with undetermined age

**Table 2:** Mean changes in primary outcome measures (counts of users per park) over time by experimental group including group differences, significance level, effect size and 95% CIs.

	Difference T1 to T2					Difference T1 to T3				
	Intervention Mean (SD)	Comparison Mean (SD)	Group Difference	*t-*Test *p*-Value	Effect Size *d* (95% CI)	Intervention Mean (SD)	Comparison Mean (SD)	Group Difference	*t-*Test *p*-Value	Effect Size *d* (95% CI)
**6 park intention-to-treat analysis**										
Park use	228.3 (92.7)	7.3 (31.5)	221.0	0.02	7.0 (2.0 to 12.0)	123.0 (138.8)	29.7 (21.5)	93.3	0.31	4.3 (−6.1 to 14.8)
Shade use (any)	46 (56.6)	−13.3 (15.0)	59.3	0.15	4.0 (−2.3 to 10.2)	44.7 (77.0)	−15.3 (32.8)	60.0	0.28	1.8 (−2.3 to 5.9)
Active ^a^	127.7 (102.3)	19.7 (15.9)	108.0	0.15	6.8 (−3.6 to 17.2)	59.3 (118.0)	43.3 (13.7)	16.0	0.83	1.2 (−12.8 to 15.1)
										
**5 park intervention received analysis ^b^**										
Park use	273 (72.1)	7.3 (31.5)	265.7	0.01	8.4 (3.9 to 12.9)	203 (11.3)	29.7 (21.5)	173.3	0.002	8.1 (5.6 to 10.6)
Shade use (any)	73.5 (43.1)	−13.3 (15.0)	86.8	0.04	5.8 (0.4 to 11.1)	75.5 (78.5)	−15.3 (32.8)	90.8	0.16	2.8 (−1.9 to 7.4)
Active ^a^	123 (144.2)	19.7 (15.9)	103.3	0.27	6.5 (−8.9 to 21.8)	118.5 (82.7)	43.3 (13.7)	75.2	0.19	2.4 (−4.9 to 16.0)

^a^ Active park visitors were observed engaging in moderate-intensity activities (i.e., walking at a casual–brisk pace) or vigorous-intensity activities (i.e., activities more vigorous than walking). ^b^ The intervention park Calder Rise Reserve did not receive the shade sail intervention and is excluded from this analysis. Note: Percentage missing T1-T3 on observation outcomes: number of park visitors 0.2%, physical activity 0.1%, unobservable for shade use T1: 11%, T2: 12%, T3: 29%. Effect sizes for T1 to T2 and T1 to T3, measured by Cohen’s d and 95% CI, were calculated as the mean group difference of intervention minus comparison parks divided by the SD for the comparison parks.

**Table 3:** Intercept survey: mean changes in secondary outcome measures over time by experimental group including group difference, significance level and 95% CIs.

	Difference T1 to T2				Difference T1 to T3			
	Intervention Mean (SD)	Comparison Mean (SD)	Group Difference	*t*-Test *p*-Value	Intervention Mean (SD)	Comparison Mean (SD)	Group Difference	*t*-Test *p*-Value
**6 park intention-to-treat analysis ^a^**								
PANAS emotional state								
Positive affect (mean)	−2.47 (2.9)	0.75 (2.7)	−3.22	0.23	−3.90 (4.4)	0.00 (0.4)	−3.90	0.20
Negative affect (mean)	−0.70 (3.1)	0.04 (0.6)	−0.75	0.70	−2.75 (4.2)	0.39 (1.1)	−3.14	0.28
Park facilities—mean attractiveness rating	3.92 (2.5)	−0.40 (2.3)	4.31	0.10	3.51 (2.6)	1.03 (1.1)	2.48	0.20
Community social cohesion score ^b^	-	-	-	-	1.04 (5.6)	−2.62 (1.8)	3.66	0.34
Frequency met/talked to new people at park in past 3 months	−0.31 (0.7)	−0.44 (0.6)	0.13	0.82	0.39 (0.9)	−1.2 (0.6)	1.62	0.05
Frequency met/talked to known people at park in past 3 months	−0.43 (0.8)	−0.53 (0.7)	0.10	0.87	0.60 (1.8)	−0.88 (1.1)	1.48	0.28
Frequency participated in social event at park in past 3 months	−0.05 (0.5)	−0.47 (0.8)	0.41	0.49	0.41 (1.0)	0.04 (0.5)	0.37	0.58

^a^ Park visitors’ self-reported emotional state, frequency of social engagement, and park aesthetic rating as measured in park intercept surveys at each study time-period. ^b^ Survey questions for community social cohesion score were not asked at T2. Instead the set of five questions asked if compared to last spring/summer, I feel less, about the same or more about the following statements to do with my community. Note: Number of intercept surveys completed: (i) overall T1 = 88, T2 = 103, T3 = 66; (ii) by study group T1: Ix = 40, C = 48; T2: Ix = 78, C = 25; T3: Ix = 43, C = 23. Percentage missing T1–T3 on self-reported outcomes: positive affect 28%, negative affect 32%, park aesthetics score 20%, community social cohesion score 10%, frequency met/talked to new people 2%, frequency met/talked to known people 3%, frequency participated in social event 2%.

**Table 4:** Total person counts ^a^ of primary outcomes for individual parks by study period and matched pair ^b^.

		T1 (n = 1670)			T2 (n = 2377)			T3 (n = 2128)		
Outcome		Pair A	Pair B	Pair C	Pair A	Pair B	Pair C	Pair A	Pair B	Pair C
Park use	Intervention	214	96	536	436	420	675	425	291	499
	Comparison	332	176	316	305	211	330	337	220	356
Physical activity (active)	Intervention	193	80	460	214	305	597	253	257	401
	Comparison	215	145	270	248	147	294	273	186	301
Shade use (any)	Intervention	0	2	35	104	45	26	131	22	18
	Comparison	24	65	1	12	36	2	24	12	8

^a^ The total person counts consisted of aggregated data from 11 half-hourly observation scans across the day on each of eight dates during the study period. Note the total counts for Pair C’s comparison park had one missed observation scan (late afternoon) on one date at T3. ^b^ Pair A: Ix—Dalton Reserve and C—Lowe Crescent; Pair B: Ix-Wahgunyah Reserve and C—Cowper Reserve; Pair C: Ix—Calder Rise Reserve and C—International gardens.

## Appendix A. Study Weather Conditions

**Table A1:** Mean ambient air temperature as recorded ^a^ during observation times in each study period.

Time of Day	T1 Mean (min—max) °C	T2 Mean (min—max) °C	T3 Mean (min—max) °C
7 am–8:30 am	15.4	15.4	15.7
	(7.9–31.5)	(8.1–26.4)	(9.1–26.4)
11:30 am–1 pm	23.5	19.4	24.0
	(15.9–39.8)	(14.0–30.3)	(17.4–29.7)
4 pm–6:30 pm	22.3	21.3	26.7
	(15.2–31.4)	(15.3–34.7)	(16.8–32.7)

^a^ Temperature recorded at the Bureau of Meteorology Melbourne Airport weather station (ID: 086282, Lat: −37.67 Lon: 144.83 a distance of approximately 13 to 22 km from the study parks). Note: Only four rainfall events were recorded at the Melbourne Airport weather stations during the study observation dates. The precipitation recorded during each study period was: T1: 0.2 mm on Nov 30, 7:00 am to 8:30 am; 2 mm on Dec 11th, 4:00 pm to 6:30 pm. T2: 4 mm on Dec 6, 7:00 am to 8:30 am. T3: 0.2 mm on Feb 4, 4:00 pm to 6:30 pm).

## References

[1] Lee I.M., Shiroma E.J., Lobelo F., Puska P., Blair S.N., Katzmarzyk P.T. (2012). Lancet Physical Activity Series Working Group. Effect of physical inactivity on major non-communicable diseases worldwide: An analysis of burden of disease and life expectancy. Lancet.

[2] Kyu H.H., Bachman V.F., Alexander L.T., Mumford J.E., Afshin A., Estep K., Veerman J.L., Delwiche K., Iannarone M.L., Moyer M.L. (2016). Physical activity and risk of breast cancer, colon cancer, diabetes, ischemic heart disease, and ischemic stroke events: Systematic review and dose-response meta-analysis for the Global Burden of Disease Study 2013. BMJ.

[3] Bedimo-Rung A.L., Mowen A.J., Cohen D.A. (2005). The significance of parks to physical activity and public health: A conceptual model. Am. J. Prev. Med..

[4] Schipperijn J., Cerin E., Adams M.A., Reis R., Smith G., Cain K., Christiansen L.B., van Dyck D., Gidlow C., Frank L.D. (2017). Access to parks and physical activity: An eight country comparison. Urban For. Urban Green.

[5] Liu H., Li F., Li J., Zhang Y. (2017). The relationships between urban parks, residents’ physical activity, and mental health benefits: A case study from Beijing, China. J. Environ. Manag..

[6] Ward J.S., Duncan J.S., Jarden A., Stewart T. (2016). The impact of children’s exposure to greenspace on physical activity, cognitive development, emotional wellbeing, and ability to appraise risk. Health Place.

[7] Sugiyama T., Leslie E., Giles-Corti B., Owen N. (2008). Associations of neighbourhood greenness with physical and mental health: Do walking, social coherence and local social interaction explain the relationships?. J. Epidemiol. Community Health.

[8] Cohen D.A., Marsh T., Williamson S., Golinelli D., McKenzie T.L. (2012). Impact and cost-effectiveness of family fitness zones: A natural experiment in urban public parks. Health Place.

[9] Lachowycz K., Jones A.P., Page A.S., Wheeler B.W., Cooper A.R. (2012). What can global positioning systems tell us about the contribution of different types of urban greenspace to children’s physical activity?. Health Place.

[10] Engelberg J.K., Conway T.L., Geremia C., Cain K.L., Saelens B.E., Glanz K., Frank L.D., Sallis J.F. (2016). Socioeconomic and race/ethnic disparities in observed park quality. BMC Public Health.

[11] Crawford D., Timperio A., Giles-Corti B., Ball K., Hume C., Roberts R., Andrianopoulos N., Salmon J. (2008). Do features of public open spaces vary according to neighbourhood socio-economic status?. Health Place.

[12] Anderson C., Jackson K., Egger S., Chapman K., Rock V. (2014). Shade in urban playgrounds in Sydney and inequities in availability for those living in lower socioeconomic areas. Aust. N. Z. J. Public Health.

[13] Ellaway A., Kirk A., Macintyre S., Mutrie N. (2007). Nowhere to play? The relationship between the location of outdoor play areas and deprivation in Glasgow. Health Place.

[14] Mitchell R., Popham F. (2008). Effect of exposure to natural environment on health inequalities: An observational population study. Lancet.

[15] Sallis J.F., Owen N., Glanz K., Rimer B.K., Viswanath K. (2015). Ecological models of health behavior. Health Behavior: Theory, Research, and Practice.

[16] McLeroy K.R., Bibeau D., Steckler A., Glanz K. (1988). An ecological perspective on health promotion programs. Health Educ. Q..

[17] Sallis J.F., Floyd M.F., Rodríguez D.A., Saelens B.E. (2012). Role of built environments in physical activity, obesity, and cardiovascular disease. Circulation.

[18] Australian Institute of Health and Welfare (2016). Skin Cancer in Australia.

[19] Fransen M., Karahalios A., Sharma N., English D.R., Giles G.G., Sinclair R.D. (2012). Non-melanoma skin cancer in Australia. Med. J. Aust..

[20] Whiteman D.C., Green A.C., Olsen C.M. (2016). The growing burden of invasive melanoma: Projections of incidence rates and numbers of new cases in six susceptible populations through 2031. J. Investig. Dermatol..

[21] Hartig T., Evans G.W., Jamner L.D., Davis D.S., Gärling T. (2003). Tracking restoration in natural and urban field settings. J. Environ. Psychol..

[22] Dobbinson S.J., Veitch J., Salmon J., Wakefield M., Staiger P.K., MacInnis R.J., Simmons J. (2017). Study protocol for a natural experiment in a lower socioeconomic area to examine the health-related effects of refurbishment to parks including built-shade (ShadePlus). BMJ Open.

[23] Brimbank City Council The Diverse Communities of Brimbank.

[24] 202020 Vision Where are All the Trees: An Analysis of Tree Canopy Cover in Urban Australia.

[25] City of Brimbank: Community Profile Idcommunity-Demographic Resources. https://profile.id.com.au/brimbank.

[26] Dobbinson S.J., White V., Wakefield M.A., Jamsen K.M., White V., Livingston P.M., English D.R., Simpson J.A. (2009). Adolescents’ use of purpose built shade in secondary schools: Cluster randomised controlled trial. BMJ.

[27] Buller D.B., English D.R., Buller M.K., Simmons J., Chamberlain J.A., Wakefield M., Dobbinson S. (2017). Shade sails and passive recreation in public parks of Melbourne and Denver: A randomized intervention. Am. J. Public Health.

[28] Watson D., Clark L.A., Tellegen A. (1988). Development and validation of brief measures of positive and negative affect: The PANAS scales. J. Pers. Soc. Psychol..

[29] Watson D., Clark L.A. (1994). The PANAS-X: Manual for the Positive and Negative Affect. Schedule—Expanded Form.

[30] Andersen P.A., Buller D.B., Walkosz B.J., Scott M.D., Beck L., Liu X., Abbott A., Eye R. (2016). Environmental variables associated with vacationers’ sun protection at warm weather resorts in North America. Environ. Res..

[31] Hayes R.J., Moulton L.H. (2017). Cluster Randomised Trials.

[32] Dobbinson S.J., Simmons J., Chamberlain J.A., MacInnis R., Salmon J., Staiger P.K., Wakefield M., Veitch J. ShadePlus. Ann Arbor, MI: Inter-university Consortium for Political and Social Research [distributor], 18 August 2020.

[33] Hunter R.F., Christian H., Veitch J., Astell-Burt T., Hipp J.A., Schipperijn J. (2015). The impact of interventions to promote physical activity in urban green space: A systematic review and recommendations for future research. Soc. Sci. Med..

[34] Veitch J., Salmon J., Crawford D., Abbott G., Giles-Corti B., Carver A., Timperio A. (2018). The REVAMP natural experiment study: The impact of a play-scape installation on park visitation and park-based physical activity. Int. J. Behav. Nutr. Phys. Act..

[35] Roberts H., McEachan R., Margary T., Conner M., Kellar I. (2016). Identifying effective behavior change techniques in built environment interventions to increase use of green space: A systematic review. Environ. Behav..

[36] Slater S., Pugach O., Lin W., Bontu A. (2016). If you build it will they come? Does involving community groups in playground renovations affect park utilization and physical activity?. Environ. Behav..

[37] Veitch J., Ball K., Crawford D., Abbott G.R., Salmon J. (2012). Park improvements and park activity: A natural experiment. Am. J. Prev. Med..

[38] Tester J., Baker R. (2009). Making the playfields even: Evaluating the impact of an environmental intervention on park use and physical activity. Prev. Med..

[39] Cohen D.A., Marsh T., Williamson S., Han B., Derose K.P., Golinelli D., McKenzie T.L. (2014). The potential for pocket parks to increase physical activity. Am. J. Health Promot..

[40] Lal A., Moodie M., Abbott G., Carver A., Salmon J., Giles-Corti B., Timperio A., Veitch J. (2019). The impact of a park refurbishment in a low socioeconomic area on physical activity: A cost-effectiveness study. Int. J. Behav. Nutr. Phy..

[41] Combs M. (2015). Musical stairs: Encouraging Physical Activity through Persuasive Technology. Student Research. Honor Scholar. Senior Thesis.

[42] Costigan S.A., Veitch J., Crawford D., Carver A., Timperio A. (2017). A cross-sectional investigation of the importance of park features for promoting regular physical activity in parks. Int. J. Environ. Res. Public Health.

[43] King D.K., Litt J., Hale J., Burniece K.M., Ross C. (2015). ‘The park a tree built’: Evaluating how a park development project impacted where people play. Urban. For. Urban. Green..

[44] City of Brimbank: Community Profile SEIFA by Local Government Area. https://profile.id.com.au/brimbank/seifa-disadvantage.

[45] Veitch J., Flowers E., Ball K., Deforche B., Timperio A. (2020). Exploring children’s views on important park features: A qualitative study using walk-along interviews. Int. J. Environ. Res. Public Health.

[46] Veitch J., Flowers E., Ball K., Deforche B., Timperio A. (2020). Designing parks for older adults: A qualitative study using walk-along interviews. Urban For. Urban Green.

[47] Dijkstra K., Pieterse M.E., Pruyn A. (2008). Stress-reducing effects of indoor plants in the built healthcare environment: The mediating role of perceived attractiveness. Prev. Med..

[48] Sturm R., Cohen D. (2014). Proximity to urban parks and mental health. J. Ment. Health Policy Econ..

